# Clinical effects of switching from minodronate to denosumab treatment in patients with postmenopausal osteoporosis: a retrospective study

**DOI:** 10.1186/s12905-020-00913-x

**Published:** 2020-03-05

**Authors:** Masaki Kobayashi, Kenjiro Sawada, Akihiko Yoshimura, Misa Yamamoto, Aasa Shimizu, Kotaro Shimura, Naoko Komura, Mayuko Miyamoto, Kyoso Ishida, Tadashi Kimura

**Affiliations:** Department of Obstetrics and Gynecology, Osaka University Graduate School of Medicine, 2-2 Yamadaoka, Suita, Osaka, 565-0871 Japan

**Keywords:** Postmenopausal osteoporosis, Denosumab, Minodronate, Switching therapy

## Abstract

**Background:**

Denosumab is a major treatment option for patients with postmenopausal osteoporosis; however, the evidence for its use is lacking. Therefore, in this 24-month retrospective study, we aimed to evaluate the effects of switching from minodronate (MIN) to denosumab in these patients.

**Methods:**

Patients with postmenopausal osteoporosis either switched from MIN to denosumab (Group 1; *n* = 32) or continued MIN treatment (Group 2; *n* = 24). Bone mineral density (BMD) of the lumbar spine (L2–L4) and femoral neck was assessed at baseline and every 6 months for 24 months. Serum bone-specific alkaline phosphatase (BAP) and N-terminal telopeptide were measured at baseline, 12 months, and 24 months.

**Results:**

Twenty-nine of the 32 patients (90.6%) in group 1 and all patients (24/24) in group 2 completed the 24-month follow-up. Switching from MIN to denosumab (Group 1) significantly increased lumbar BMD at 12, 18, and 24 months (6.1, 7.4, and 9.6%, respectively) and femoral neck BMD at 12, 18, and 24 months (2.8, 3.2, and 3.4%, respectively), whereas MIN continuous treatment (Group 2) showed no significant difference from baseline. Switching therapy also showed a significant decrease in serum BAP from baseline to 12 and 24 months (− 19.3 and − 26.5%, respectively) and serum NTX from baseline to 12 months (− 13.1%), whereas continuous MIN treatment failed to show any significant differences from baseline.

**Conclusion:**

Switching from MIN to denosumab in patients with postmenopausal osteoporosis showed clinical benefits with regard to BMD and bone turnover markers in comparison with continuous MIN treatment. It may therefore be a valid treatment option in the clinical setting.

## Background

Postmenopausal osteoporosis is the most common bone disease. It is characterized by reduced bone mass and microscopic changes in the architecture resulting in impaired strength of bones, with consequent increased susceptibility to fracture, which results in high medical expenditures and substantial morbidity with a decrease in quality of life [1, 2]. The bone mineral density (BMD) reduces with age in the entire population, and women are particularly at higher risk since they rapidly lose bone in the peri- and post- menopausal periods [2]. Age, sex, family history, low baseline weight, weight loss, and alcohol use in women, and smoking in men are generally associated with low BMD [2]. Owing to an increasing life expectancy, the number of patients has been progressively increasing worldwide [3]. One of the most debilitating complications of osteoporosis is hip fracture, which has an estimated probability of 3.5% in men and 14.6% in women around the age of 50 years old. Approximately 20–50% patients with hip fracture suffer from a decreased quality of life, depression, loss of self-esteem, and social isolation, which leads to the drastic elevation of one-year mortality rate, up to 14–60% [4–7]. Vertebral fractures are extremely common complications of osteoporosis and often asymptomatic. However, multiple vertebral thoracic fractures may induce restrictive lung disease and secondary heart problems. Lumbar fractures may cause gastrointestinal complaints, back pain (both, acute and chronic), depression, and positional restriction, resulting in increased mortality [7, 8]. In view of these numerous complications, the main objective of treating postmenopausal osteoporosis is the prevention of future fractures.

The two types of treatment options for osteoporosis include anti-resorptive and anabolic agents. Anti-resorptive drugs include bisphosphonates (e.g. alendronate, minodronate (MIN), etidronate, risedronate, pamidronate, and zoledronate), selective estrogen-receptor modulators (raloxifene and bazedoxifene), active vitamin D3 derivatives (alfacalcidol and eldecalcitol), a fully human monoclonal antibody to receptor activator of nuclear factor κ-B ligand (RANKL; denosumab), and thyroid hormone (calcitonin). Anabolic agents include parathyroid hormone (teriparatide), parathyroid-related peptide synthetic analogs (abaloparatide), and a sclerostin inhibitor (romosozumab) [9]. Among these treatment options, bisphosphonates are the most widely used all over the world. MIN is a third-generation bisphosphonate and the strongest inhibitor of bone resorption among the currently available oral bisphosphonates. In terms of bone resorption inhibition, MIN is 1000-fold more effective than etidronate and 10–100-fold more effective than alendronate [10]. Large randomized, placebo-controlled, double-blind clinical trials revealed a significant increase in bone mineral density (BMD) of both, the lumbar spine and femoral neck over 3 years of MIN therapy and a risk reduction in vertebral fractures in Japanese women with postmenopausal osteoporosis [11]. Therefore, MIN continues to be one of the most widely used bisphosphonates in Japan.

Denosumab is a fully human monoclonal anti-RANKL IgG2 antibody that inhibits the binding of RANKL to its receptor on osteoclasts, thereby decreasing the bone-resorption activity of mature osteoclasts [12]. In the phase 3 Fracture Reduction Evaluation of Denosumab in Osteoporosis Every 6 Months (FREEDOM) trial, denosumab treatment significantly reduces the incidence of new vertebral, nonvertebral, and hip fractures compared with placebo treatment [13]. Furthermore, in the FREEDOM Extension trial, 10-year treatment with denosumab increases the BMD of patients progressively with a significantly lower incidence of fracture [14]. In Japan, denosumab is currently one of the first treatment options, as evidenced by the report that sales of denosumab exceed that of each bisphosphonate [15]. Lyu et al. performed a meta-analysis of 10 eligible trials including 5361 participants comparing denosumab and bisphosphonates [16]. Denosumab increases BMD more than bisphosphonates at the lumbar spine, total hip, and femoral neck. Furthermore, one study showed that denosumab has a lower osteoporotic fracture incidence than alendronate at 24 months (risk ratio, 0.51; 95% confidence interval, 0.27 to 0.97) [17]. Based on this clinical evidence, both denosumab and bisphosphonates are widely prescribed for osteoporosis in Japan. However, since denosumab is more easily administered by two annual injections with less complication, which may possibly increase the compliance rate, it appears to be the better choice between the two drugs. Therefore, we assumed that it can be considered as a reasonable treatment option to switch from bisphosphonates to denosumab in the clinical setting. However, only a few studies have reported the efficacy of switching from bisphosphonates to denosumab; therefore, the evidence level remains low.

In view of these findings, we advised patients treated with MIN for more than 2 years to switch to denosumab if they agreed after the detailed explanation of each drug from treating physicians. In this study, we aimed to retrospectively evaluate the treatment effects of patients who switched to denosumab and compared with those of patients who continued MIN treatment.

## Methods

### Subjects and study design

This retrospective study included patients with postmenopausal osteoporosis between January 2012 and December 2018. It was conducted in accordance with the ethical standards of the Helsinki II Declaration and approved by the institutional review board of Academic Clinical Research Center of Osaka University (No. 19130). The participants were all Japanese women aged 45–81 years old. They were naturally or surgically menopausal (> 12 months since the last menstrual period or after oophorectomy). The BMDs of their lumbar (at the L2–L4 vertebral site) and femoral neck were measured using dual-energy X-ray absorptiometry (Discovery-A; Hologic, Bedford, MA, USA). In our institution, the coefficient of variance is < 1.0% for the lumbar spine. The subjects who were diagnosed with osteoporosis (below − 2.5 standard deviations of the mean value of healthy Japanese 20–44-year-old normal women) initially received 50 mg of MIN (Astellas Pharma Inc., Tokyo, Japan) once a month with daily supplementation of 1 μg of alfacalcidol and 600 mg of calcium. They were instructed to take the tablets with 200 mL of water at least 30 min before breakfast and not to lie down within 30 min after taking the medication. After the treatment with MIN for 2 years, they were asked to switch to denosumab, if they agreed after the detailed explanation of each drug from treating physicians. A total of 32 patients chose this treatment option, whereas 24 patients wished to continue MIN treatment. Patients who switched to denosumab received 60 mg of denosumab (Daiichi Sankyo Company, Tokyo, Japan) subcutaneously every 6 months. They further took vitamin D and calcium supplementation 2 tablets (762.5 mg of precipitated calcium carbonate, 200 IU of cholecalciferol and 59.2 mg of magnesium carbonate) once daily. The information about this study was posted on the hospital homepage with waiver of informed consent. Subjects were excluded for the following reasons: 1) hypocalcemia, 2) severe diabetes, 3) thyroid or parathyroid disease, 4) hepatic disease, 5) malignant disease, 6) hormone replacement therapy, 7) significantly impaired renal function (creatinine clearance < 35 mL/min as estimated by the Cockcroft and Gault method), 8) hypersensitivity to denosumab, 9) concomitant use of other agents for osteoporosis, 10) concomitant use of agents potentially affecting bone turnover, and 11) inappropriate to participate in the study for other reasons. None of the participants had previously received any bisphosphonates other than MIN; however, patients who had previously received hormone replacement therapy and/or selective estrogen-receptor modulators were included in this study.

### Study assessments

DEXA (Discovery-A; Hologic, Bedford, MA, USA) scans were obtained at baseline, 6, 12, 18, and 24 months for the lumbar spine (L2–L4) on posteroanterior projections and the femoral neck. The diagnosis of osteoporosis was made in accordance with the 2012 version of the Japanese diagnostic criteria published by the Japanese Society for Bone and Mineral Research [18], and the BMD values for the L2–L4 spine widely used in Japan, were employed. Each percent change from the baseline was calculated, and the data were shown as mean value ± SE.

With regard to biochemical bone markers, serum type I collagen cross-linked N-terminal telopeptide (NTX) was measured as the bone absorptive parameter and serum bone-specific alkaline phosphatase (BAP) as the bone formation marker. At baseline, 12, and 24 months, serum NTX was measured using enzyme-linked immunosorbent assay (SRL Inc., Tokyo, Japan; normal range for women: 7.5–16.5 nmol BCE/L [≤45 years old], 10.7–24.0 nmol BCE/L [> 45 years old]), and serum BAP was measured using chemiluminescent enzyme immunoassay (SRL Inc., Tokyo, Japan; normal range for women: 2.9–14.5 μg/L [≤45 years old], 3.8–22.6 μg/L [> 45 years old]).

Adverse events were recorded at baseline and all post visits, and their severity and relationship to treatment were evaluated by the investigators.

### Endpoints

The primary efficacy endpoint was the percent change in lumbar BMD and femoral neck BMD from baseline. The secondary efficacy endpoints were the percent change from the baseline in serum BAP and NTX. Safety endpoints included adverse events.

### Statistical analysis

Paired Student’s *t*-test was performed to make baseline comparisons of BMD and bone turnover markers. Each parameter was compared between groups using the non-parametric Wilcoxon signed-rank test. Changes in BMD and bone turnover markers from baseline to specified time points within each study group were also compared using the non-parametric Wilcoxon signed-rank test. Results are expressed as the mean ± standard error. *P* value < 0.05 indicated statistical significance. All tests were performed using JMP Pro 10 (SAS Institute Inc., Cary, NC, USA).

## Results

### Characteristics of subjects

**Fig. 1:**
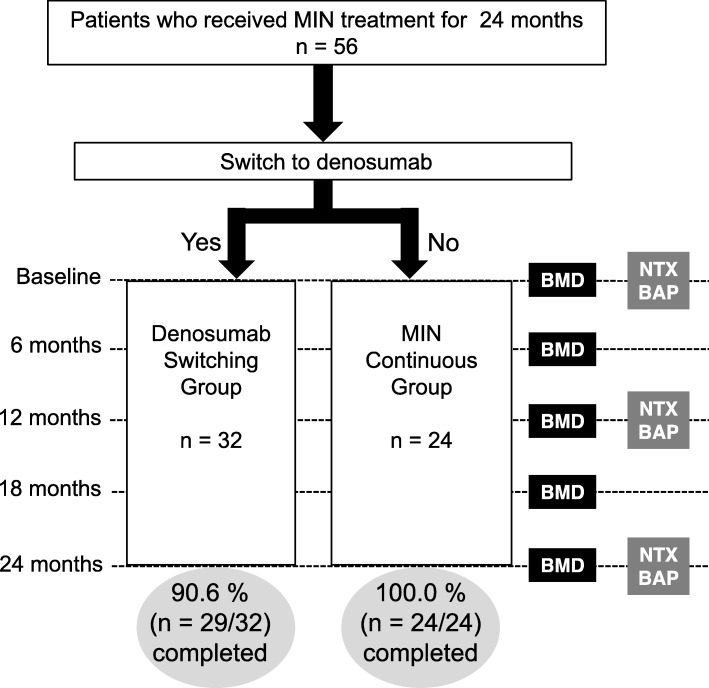
Study design and schedule. Patients after minodronate (MIN) treatment for 2 years were asked for their willingness to switch to denosumab. Bone mineral density (BMD) and bone turnover markers (NTX and BAP) were evaluated as indicated. Adverse events were also recorded at baseline and all post visits

The outline of this retrospective study is summarized in Fig. 1. A total of 56 patients who were initially treated with MIN were retrospectively analyzed. Among them, 46 (82%) were older than 65 years of age and the remaining 10 were younger than 65 years. Patients older than 65 years of age received DEXA scans to evaluate the presence of osteoporosis. Among the remaining 10, 8 had underwent salpingo-oohorectomy, 1 had received an aromatase inhibitor for breast cancer, and 1 had received hematopoietic stem cell transplantation for leukemia. Since these previous treatments are obvious risk factors for osteoporosis, they received DEXA scans for check-up. Among them, 32 (57.1%) switched to denosumab (group 1) and 24 (42.9%) continued MIN treatment after a 2-year initial treatment (group 2). The characteristics of both groups are summarized in Table 1. The median age of all the participants was 69 years (range, 45–81 years), and the mean body mass index was 21.2 ± 2.5. These two groups did not differ with respect to age, height, weight, body mass index, time since menopause, percentage of patients who had experienced surgical menopause, femoral neck BMD, lumbar BMD, serum NTX, and serum BAP. During the treatment period, no patient experienced new clinical fractures. In group 1, 29 of the 32 (90.6%) patients completed the 24-month follow-up. Three patients discontinued denosumab treatment because two had adverse events (itching without rash and repeated leg cramps, respectively), and one was lost to follow-up. In group 2, all (24/24) patients completed the 24-month follow-up.

**Table 1:** Characteristics of the subjects

Characteristics	Switching group (***n*** = 32)	Continuing group (***n*** = 24)	***P*** value
Age (years), median (range)	72 (47–81)	68 (45–80)	0.152
Height (cm)	151.3 ± 4.0	152.9 ± 5.0	0.318
Weight (kg)	49.6 ± 6.1	48.3 ± 6.0	0.425
Body mass index (kg/m^2^)	21.7 ± 2.6	20.7 ± 2.3	0.208
Time since menopause (year)	19.2 ± 7.8	14.6 ± 9.3	0.097
Number of cases with surgical menopause, *n* (%)	9 (28)	5 (21)	0.756
Femoral neck BMD (g/cm^2^)	0.517 ± 0.064	0.553 ± 0.077	0.094
Femoral neck BMD (T-score)	−2.2 ± 0.3	−2.0 ± 0.3	0.076
Lumbar BMD (g/cm^2^)	0.714 ± 0.096	0.744 ± 0.083	0.175
Lumbar BMD (T-score)	−1.9 ± 0.4	−1.7 ± 0.5	0.155
Serum NTX (nmol BCE/L)	12.36 ± 3.62	13.35 ± 6.87	0.984
Serum BAP (U/L)	9.63 ± 3.13	9.10 ± 2.57	0.620

Mean ± Standard Error (SE), unless otherwise noted

*BMD* bone mineral density, *NTX* N-terminal telopeptide, *BAP* bone-specific alkaline phosphatase

### Bone mineral density

**Fig. 2:**
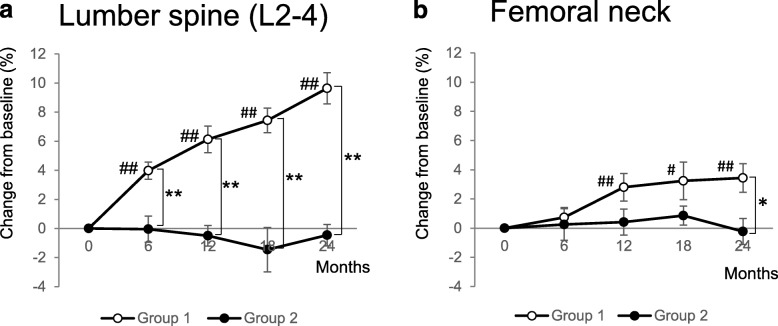
Percent changes from baseline in bone mineral density (BMD) at the lumbar spine (**a**) and femoral neck (**b**). Open and closed circles indicate the switching group (group 1) and continuous MIN group (group 2), respectively. Data are shown as mean ± standard error (SE). ^#^*P* < 0.05, ^##^*P* < 0.01 change from baseline within each treatment group. *P < 0.05, **P < 0.01 Group 1 versus Group 2

Percent changes of lumbar BMD from baseline to 6, 12, 18, and 24 months are shown in Fig. 2a. Switching from MIN to denosumab (Group 1) significantly increased lumbar BMD at 12, 18, and 24 months (6.1, 7.4, and 9.6%, respectively), whereas continuous MIN treatment (group 2) showed no significant difference from baseline to any specified points (− 0.5, − 1.5, and − 0.5% at 12, 18, and 24 months, respectively). Accordingly, a significant difference was found between both groups at each time point. With respect to femoral neck BMD, switching from MIN to denosumab (Group 1) significantly increased femoral neck BMD at 12, 18, and 24 months (2.8, 3.2, and 3.4%, respectively), whereas continuous MIN treatment (group 2) failed to show any significant differences from baseline to any specified points (0.4, 0.9, and − 0.2% at 12, 18, and 24 months, respectively) (Fig. 2b). In comparison with both groups, a significant difference was observed at 24 months (3.4 vs − 0.2%; *P* < 0.05).

### Bone turnover markers

**Fig. 3:**
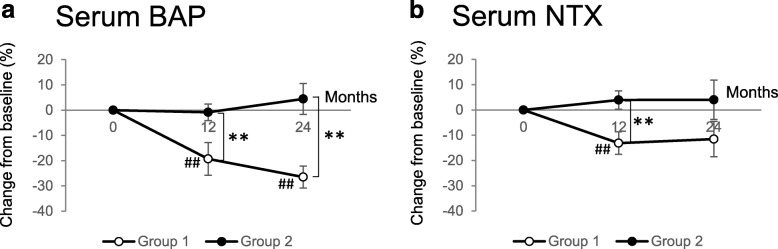
Percent changes from baseline in the serum concentration of bone turnover markers, BAP (**a**) and NTX (**b**). Open and closed circles indicate the switching group (group 1) and continuous MIN group (group 2), respectively. Data are shown as mean ± standard error. ^##^*P* < 0.01 change from baseline within each treatment group. **P < 0.01 group 1 versus group 2

Percent changes in bone turnover markers from baseline are shown in Fig. 3. Switching from MIN to denosumab (group 1) showed a significant decrease from baseline to 12 and 24 months in serum BAP (− 19.3 and − 26.5%, respectively (Fig. 3a)). For serum NTX, group 1 showed a significant decrease at 12 months (− 13.1%) (Fig. 3b). In contrast, continuous MIN treatment (group 2) failed to show any significant difference at any specified points not only in serum BAP but also in NTX. Comparison of both groups showed that group 1 had a significantly greater decrease than group 2 in serum BAP at 12 months (− 19.3 vs − 0.8%; *P* < 0.01) and 24 months (− 26.5 vs 4.4%; *P* < 0.01) and in serum NTX at 12 months (− 13.1 vs 3.9%; *P* < 0.01).

### Adverse events

In total, three cases of adverse events were reported: 2 cases in group 1 and 1 in group 2. In group 1, one presented itching without rash and the other presented repeated leg cramps. Both stopped receiving denosumab treatment after two cycles. Serum calcium concentrations of the subject who experienced repeated leg cramps was 9.4 and 9.2 mg/dl (normal range; 8.4–10.2 mg/dl), at 1 and 6 months after switching to denosumab, respectively. One case in group 2 had broken her right little finger; however, she continued MIN treatment. No serious adverse events, such as hypocalcemia and osteoporosis-related fractures, were reported during the follow-up period.

## Discussion

In this retrospective study, switching from MIN to denosumab significantly increased lumbar BMD (9.6% at 24 months) and femoral neck BMD (3.4% at 24 months), with a certain decrease in bone turnover markers. This indicates that instead of continuing with MIN, patients could switch to denosumab, which is more effective at improving BMD. Regarding bone turnover markers, switching from MIN to denosumab significantly decreased serum BAP from baseline to 12 and 24 months (− 19.3 and − 26.5%, respectively); however, while it significantly decreased serum NTX (− 13.1%) at 12 months no corresponding decrease (− 11.5%) was observed at 24 months. Circadian rhythms strongly affect the variability of bone turnover markers, and greater circadian changes are seen in bone resorption markers than bone formation markers [19]. Since it was not practically possible to collect serum samples at a fixed time from outpatients, the collected samples needed to be validated, particularly for NTX; this could explain the lack of significant decrease in serum NTX at 24 months.

Although both bisphosphonates including MIN and denosumab are antiresorptive drugs for osteoporosis, pharmacological mechanisms of action and effects differ [20]. Meta-analyses comparing these two drugs indicate an overall greater efficacy of denosumab on bone metabolism [16, 21]. Several mechanisms may explain the differential impacts. Bisphosphonates bind to bone mineral and are taken up by mature osteoclasts at bone resorption sites. Thereafter, bisphosphonates impair the resorptive function of osteoclasts, but these disabled osteoclasts may persist. In contrast, denosumab binds to RANKL and thereby blocks osteoclast formation, function, and survival [20], which causes more potent and rapid inhibitory effects on bone resorption. With regard to pharmacokinetics, denosumab seems to result in the recovery of bone remodeling capability at the end of each therapy cycle [22]. New spaces of bone tissue go through the process of bone remodeling but fail to undergo resorption under the inhibitory impact of the next dose of denosumab [23]. In addition, several studies have raised concerns about the potential harm or decreased efficacy of long-term bisphosphonate use on fracture risks [24, 25]. Therefore, denosumab may lead to a more favorable positive balance in bone metabolism compared with bisphosphonates. In view of these findings, switching from a long-term bisphosphonate therapy to denosumab appears reasonable in the clinical setting.

In general, patients with osteoporosis need to receive their treatment adherently according to the dosing instructions and for the prescribed duration, as it is evident that good compliance of osteoporosis treatment is important for reducing the risk of fracture [26]. In their meta-analysis, Karlsson et al. found that the persistence with denosumab in women with postmenopausal osteoporosis is approximately two-fold higher than pooled persistence rates from a meta-analysis of retrospective data on oral bisphosphonates [26]. Considering the relatively lower persistence with oral bisphosphonates, switching to another treatment option might be acceptable if patients do not acquire estimated effects on bone metabolism after a certain period of oral bisphosphonate treatment.

Several studies have shown the validity of switching therapies. Recknor et al. found that denosumab treatment for osteoporosis results in greater BMD increases than ibandronate treatment at all measured sites in postmenopausal women previously treated with bisphosphonates [27]. Roux et al. indicated that transitioning to denosumab is well tolerated and more effective than risedronate in reducing bone turnover and increasing BMD in postmenopausal women who had been suboptimally adherent to alendronate therapy [28]. Kendler et al. reported that transition to denosumab produces greater increases in BMD at all measured skeletal sites and a greater reduction in bone turnover than continued alendronate [29]. In this study, we first revealed that switching from MIN, which is a third-generation bisphosphonate and much more active for the inhibition of bone resorption than alendronate or risedronate [30–32], to denosumab showed similar beneficial effects on BMD and bone turnover markers. Since MIN is one of the most widely used bisphosphonates in Japan [15], establishing clinical evidence for switching therapy from MIN would benefit patients.

Two adverse events (leg cramp and itching without rash) in the switching group occurred within a few months after the second dose of denosumab. Leg cramps can be due to hypocalcemia, but the serum calcium concentration of the subject was within normal range. Considering the short time onset, they may be not related to denosumab use. Possible severe adverse events that may occur after long-term bisphosphonate or denosumab use are atypical femoral fracture and osteonecrosis of the jaw [33, 34]; however, no subjects in this study presented those complications. Considering the low incidences of complications, a larger number of participants with longer treatment period are necessary to prove the safety and feasibility of the switching therapy. Recently, Miller et al. reported a pooled analysis to estimate the efficacy and safety of switching to denosumab vs. continuing bisphosphonate treatment in 2850 postmenopausal women who previously received oral bisphosphonates [35]. In this study, the incidence rate of adverse events leading to drug discontinuation was higher in the bisphosphonate continuing vs. the denosumab switching group (4.0 vs. 1.8%), suggesting the feasibility of the switching therapy. The bisphosphonate group discontinued their treatment due to adverse events, most commonly arthralgia, ameloblastoma, and cerebral ischemia, although it is believed that the overall safety profiles of the bisphosphonate and denosumab were similar. No events of osteonecrosis of the jaw were reported; however, a total of 3 patients had experienced atypical femoral fractures, and all of those had long previous exposure to oral bisphosphonates (an average of 6 years) [35].

This study has certain limitations. It was a retrospective study involving a small number of patients, and the patients were not randomized. It is pivotal to make an appropriate control to prove the efficacy, which we were unable to perform. However, sample size was calculated using Power and Sample Size Calculation (http://biostat.mc.vanderbilt.edu/wiki/Main/PowerSampleSize). When a power of 80% and level α = 0.05 were set, 180 experimental subjects and 135 control subjects are needed to reject the null hypothesis that the population means of the experimental and control groups are equal with probability (power) 0.8. No evident fractures were observed in patients during the study period, probably owing to the considerably small sample size and short observation period. Further studies are warranted to confirm the clinical benefits of switching therapy.

## Conclusions

In women with postmenopausal osteoporosis who had taken MIN for a certain period, switching to denosumab was effective in increasing the BMD of the femoral neck and lumbar spine with a decrease in bone turnover markers. Postmenopausal women with osteoporosis may switch from MIN to denosumab, which is more effective at improving BMD than continuous MIN. Switching of therapy may be considered as one of the effective treatment options.

## Data Availability

The datasets used and/or analyzed during the current study are available from the corresponding author on reasonable request.

## Abbreviations

BAP: Bone-specific alkaline phosphatase
BMD: Bone mineral density
MIN: Minodronate
NTX: Type I collagen cross-linked N-terminal telopeptide
RANK: Receptor activator of nuclear factor-κB
RANKL: Receptor activator of nuclear factor κ-B ligand

## References

[1] Pavone V, Testa G, Giardina SMC, Vescio A, Restivo DA, Sessa G (2017). Pharmacological therapy of osteoporosis: a systematic current review of literature. Front Pharmacol.

[2] Das S, Crockett JC (2013). Osteoporosis - a current view of pharmacological prevention and treatment. Drug Des Devel Ther.

[3] Reid IR (2015). Short-term and long-term effects of osteoporosis therapies. Nat Rev Endocrinol.

[4] Holt G, Smith R, Duncan K, Hutchison JD, Gregori A, Reid D (2012). Outcome after sequential hip fracture in the elderly. J Bone Joint Surg Am.

[5] Cumming RG, Klineberg R, Katelaris A (1996). Cohort study of risk of institutionalisation after hip fracture. Aust N Z J Public Health.

[6] Keene GS, Parker MJ, Pryor GA (1993). Mortality and morbidity after hip fractures. BMJ..

[7] Sözen T, Özışık L, Başaran NÇ (2017). An overview and management of osteoporosis. Eur J Rheumatol.

[8] Varacallo MA, Fox EJ (2014). Osteoporosis and its complications. Med Clin North Am.

[9] Anagnostis P, Gkekas NK, Potoupnis M, Kenanidis E, Tsiridis E, Goulis DG (2019). New therapeutic targets for osteoporosis. Maturitas.

[10] Tanishima S, Morio Y (2013). A review of minodronic acid hydrate for the treatment of osteoporosis. Clin Interv Aging.

[11] Matsumoto T, Hagino H, Shiraki M, Fukunaga M, Nakano T, Takaoka K (2009). Effect of daily oral minodronate on vertebral fractures in Japanese postmenopausal women with established osteoporosis: a randomized placebo-controlled double-blind study. Osteoporos Int.

[12] Miyazaki T, Tokimura F, Tanaka S (2014). A review of denosumab for the treatment of osteoporosis. Patient Prefer Adherence.

[13] Cummings SR, San Martin J, McClung MR, Siris ES, Eastell R, Reid IR (2009). Denosumab for prevention of fractures in postmenopausal women with osteoporosis. N Engl J Med.

[14] Bone HG, Wagman RB, Brandi ML, Brown JP, Chapurlat R, Cummings SR (2017). 10 years of denosumab treatment in postmenopausal women with osteoporosis: results from the phase 3 randomised FREEDOM trial and open-label extension. Lancet Diabetes Endocrinol.

[15] News A (2019). Personal pages of answers News.

[16] Lyu H, Jundi B, Xu C, Tedeschi SK, Yoshida K, Zhao S (2019). Comparison of denosumab and bisphosphonates in patients with osteoporosis: a meta-analysis of randomized controlled trials. J Clin Endocrinol Metab.

[17] Nakamura T, Matsumoto T, Sugimoto T, Hosoi T, Miki T, Gorai I (2014). Clinical trials express: fracture risk reduction with denosumab in Japanese postmenopausal women and men with osteoporosis: denosumab fracture intervention randomized placebo controlled trial (DIRECT). J Clin Endocrinol Metab.

[18] Soen S, Fukunaga M, Sugimoto T, Sone T, Fujiwara S, Endo N (2013). Diagnostic criteria for primary osteoporosis: year 2012 revision. J Bone Miner Metab.

[19] Blumsohn A, Herrington K, Hannon RA, Shao P, Eyre DR, Eastell R (1994). The effect of calcium supplementation on the circadian rhythm of bone resorption. J Clin Endocrinol Metab.

[20] Baron R, Ferrari S, Russell RG (2011). Denosumab and bisphosphonates: different mechanisms of action and effects. Bone..

[21] Miller PD, Pannacciulli N, Brown JP, Czerwinski E, Nedergaard BS, Bolognese MA (2016). Denosumab or zoledronic acid in postmenopausal women with osteoporosis previously treated with oral bisphosphonates. J Clin Endocrinol Metab.

[22] Augoulea A, Tsakonas E, Triantafyllopoulos I, Rizos D, Armeni E, Tsoltos N (2017). Comparative effects of denosumab or bisphosphonate treatment on bone mineral density and calcium metabolism in postmenopausal women. J Musculoskelet Neuronal Interact.

[23] Hanley DA, Adachi JD, Bell A, Brown V (2012). Denosumab: mechanism of action and clinical outcomes. Int J Clin Pract.

[24] Drieling RL, LaCroix AZ, Beresford SAA, Boudreau DM, Kooperberg C, Chlebowski RT (2016). Long-term oral bisphosphonate use in relation to fracture risk in postmenopausal women with breast cancer: findings from the Women's Health Initiative. Menopause..

[25] Drieling RL, LaCroix AZ, Beresford SAA, Boudreau DM, Kooperberg C, Chlebowski RT (2017). Long-term oral bisphosphonate therapy and fractures in older women: the women's health initiative. J Am Geriatr Soc.

[26] Karlsson L, Lundkvist J, Psachoulia E, Intorcia M, Ström O (2015). Persistence with denosumab and persistence with oral bisphosphonates for the treatment of postmenopausal osteoporosis: a retrospective, observational study, and a meta-analysis. Osteoporos Int.

[27] Recknor C, Czerwinski E, Bone HG, Bonnick SL, Binkley N, Palacios S (2013). Denosumab compared with ibandronate in postmenopausal women previously treated with bisphosphonate therapy: a randomized open-label trial. Obstet Gynecol.

[28] Roux C, Hofbauer LC, Ho PR, Wark JD, Zillikens MC, Fahrleitner-Pammer A (2014). Denosumab compared with risedronate in postmenopausal women suboptimally adherent to alendronate therapy: efficacy and safety results from a randomized open-label study. Bone.

[29] Kendler DL, Roux C, Benhamou C, Brown J, Lillestol M, Siddhanti S (2010). Effects of denosumab on bone mineral density and bone turnover in postmenopausal women transitioning from alendronate therapy. J Bone Miner Res.

[30] Chatani Y (2005). Minodronic acid hydrate as a new therapeutic agent for osteoporosis. Clin Calcium.

[31] Dunford JE, Thmpson K, Coxon FP, Luckman SP, Hahn FM, Poulter CD (2001). Structure-activity relationships for inhibition of farnesyl diphosphate synthase in vitro and inhibition of bone resorption in vivo by nitrogen-containing bisphosphonates. J Pharmacol Exp Ther.

[32] Ohishi T, Matsuyama Y (2018). Minodronate for the treatment of osteoporosis. Ther Clin Risk Manag.

[33] Silverman S, Kupperman E, Bukata S (2018). Bisphosphonate-related atypical femoral fracture: managing a rare but serious complication. Cleve Clin J Med.

[34] Kuroshima S, Sasaki M, Sawase T (2019). Medication-related osteonecrosis of the jaw: a literature review. J Oral Biosci.

[35] Miller P.D., Pannacciulli N., Malouf-Sierra J., Singer A., Czerwiński E., Bone H.G., Wang C., Huang S., Chines A., Lems W., Brown J.P. (2019). Efficacy and safety of denosumab vs. bisphosphonates in postmenopausal women previously treated with oral bisphosphonates. Osteoporosis International.

